# A Novel Approach of a Low-Cost UWB Microwave Imaging System with High Resolution Based on SAR and a New Fast Reconstruction Algorithm for Early-Stage Breast Cancer Detection

**DOI:** 10.3390/jimaging8100264

**Published:** 2022-09-28

**Authors:** Ibtisam Amdaouch, Mohamed Saban, Jaouad El Gueri, Mohamed Zied Chaari, Ana Vazquez Alejos, Juan Ruiz Alzola, Alfredo Rosado Muñoz, Otman Aghzout

**Affiliations:** 1 Department of Computer Science Engineering, Système d’Information et Gènie Logiciel-Lab, École Nationale des Science Appliquèes (ENSA), University Abdelmalek Essaadi, Tetouan 93153, Morocco; 2 Department of Electronic Engineering, Escola Tècnica Superior d’Enginyeria (ETSE), University Valencia, Av. Universitat, 46100 Burjassot, Spain; 3 Department of Qatar Scientific Club, Fabrication Lab, Doha 9769, Qatar; 4 atlanTTic, Sistemas Radio, University of Vigo, 36310 Vigo, Spain; 5 Department of Señales y Comunicaciones, University of Las Palmas de Gran Canaria, 35001 Las Palmas, Spain

**Keywords:** specific absorption rate, microwave imaging, breast cancer detection, Vivaldi antenna, image reconstruction, confocal algorithm

## Abstract

In this article, a new efficient and robust approach—the high-resolution microwave imaging system—for early breast cancer diagnosis is presented. The core concept of the proposed approach is to employ a combination of a newly proposed delay-and-sum (DAS) algorithm and the specific absorption rate (SAR) parameter to provide high image quality of breast tumors, along with fast image processing. The new algorithm enhances the tumor response by altering the parameter referring to the distance between the antenna and the tumor in the conventional DAS matrices. This adjustment entails a much clearer reconstructed image with short processing time. To achieve these aims, a high directional Vivaldi antenna is applied around a simulated hemispherical breast model with an embedded tumor. The detection of the tumor is carried out by calculating the maximum value of SAR inside the breast model. Consequently, the antenna position is relocated near the tumor region and is moved to nine positions in a trajectory path, leading to a shorter propagation distance in the image-creation process. At each position, the breast model is illuminated with short pulses of low power waves, and the back-scattered signals are recorded to produce a two-dimensional image of the scanned breast. Several simulations of testing scenarios for reconstruction imaging are investigated. These simulations involve different tumor sizes and materials. The influence of the number of antennas on the reconstructed images is also examined. Compared with the results from the conventional DAS, the proposed technique significantly improves the quality of the reconstructed images, and it detects and localizes the cancer inside the breast with high quality in a fast computing time, employing fewer antennas.

## 1. Introduction

The early detection of breast cancer is vital for the disease’s treatment. It has been proven to be cost-effective at reducing morbidity and mortality. X-ray mammography is currently the gold-standard method for breast cancer detection. However, it has the disadvantages of harmful radiation, uncomfortable compression of the breast during examination, and relatively high false-negative rates [1,2]. Recently, confocal microwave imaging has attracted the interest of a number of research groups as an effective alternative.

The approach of confocal microwave imaging (CMI) was first proposed by Hagness et al. [3,4] and Fear et al. [5] for breast cancer detection. It has been adopted for UWB microwave imaging due to its validity, simplicity, robustness, and short computation time [6]. CMI is based on several assumptions regarding the dielectric properties of normal and malignant breast tissue. One of these assumptions is that the breast is primarily dielectrically homogeneous, and there is a contrast between normal and malignant breast tissue. The key signal processing component of any CMI-based breast-imaging system is the image reconstruction algorithm, which can impact the efficacy of CMI in detecting breast cancer. The performance of image-reconstruction algorithms has often been evaluated in the literature using a selective set of beam-forming algorithms [7], anatomically and dielectrically inaccurate numerical phantoms [8,9,10], and an idealized artifact removal algorithm while ignoring the impact of realistic artifact removal [11,12,13].

To construct radar images, various image reconstruction algorithms have been proposed by multiple research groups in order to improve the tumor response [14,15,16,17,18]. They have been sorted into two categories: data-independent (DI) beamforming and data-adaptive (DA) beamforming algorithms [19].

One of the most promising DI algorithms considered in microwave imaging is delay-and-sum (DAS). It involves transmitting an ultra-wideband (UWB) pulse into the breast under study, from various antenna locations encircling the breast. Nevertheless, it is a blind and non-adaptive beam-former that results in low image resolution with high levels of side lobe [13]. This instigates the development of other sophisticated methods to overcome these drawbacks.

In this article, we propose a new reconstruction algorithm approach for high-accuracy UWB microwave confocal imaging system that uses a specific absorption rate (SAR) to infer the locations of the most energy-absorbing tissue such as tumors. The new algorithm involves improving the DAS confocal algorithm by adjusting the distance parameter between antenna and tumor in the synthetic focusing matrices of DAS algorithm. This alteration aims to detect tumors inside the female breast with high accuracy and to provide high image quality. In addition, it will reduce the size of matrices and hence reduce the computational time of image processing. In this work, we have worked in a simulation environment under a CST MICROWAVE Studio Simulator to prove the efficiency of the proposed simulated imaging system. We adopted this technique, which proved to be effective in theory and was performed by many authors in the literature [20,21,22,23]. To date, this technique is still theoretical. In practice, the breast phantom will be placed in the SAR measurement testing machine to calculate SAR maximum values and detect tumor location, as described in [24,25,26]. After using the SAR testing, the phantom will be moved to the imaging system, where reconstructed images of tumor will be obtained by positioning antennas next tumor area.

In order to detect tumor location, a high-directional Vivaldi antenna is applied to a simulated cancerous breast model. At the beginning, the antenna is located at a random position around the breast at a distance of 10 mm to calculate the maximum value of SAR. As reported in [22,23,27], the coordinates of this value actually point exactly to the coordinates of tumor. Thereafter, the Vivaldi antenna position is relocated to where the tumor exists, resulting in a smaller distance between the transmitting antenna and tumor. This process is repeated for nine positions, covering a 90° angle scan of the breast, with an angular separation of 10°. At each position, the antenna transmits short EM pulses into the breast tissue in a monostatic mode. The foremost advantage of this particular method is that these signals will have a shorter propagation distance in the image-creation process, and thus they will be less affected by the attenuation and phase effects. The reflected signals are then collected by the same antenna and imported to MATLAB to find regions of dielectric scatterings inside the breast. Several simulations are conducted in order to evaluate the performance of the proposed microwave-imaging method. Different sizes and permittivities of tumors are investigated to prove its effectiveness. The reconstructed images are also examined using different numbers of antennas. The proposed approach is compared to the conventional DAS using the simulated data produced by the developed radar system. Based on the imaging results, positioning the antenna near the tumor location provides effectively high image quality. Furthermore, the quality of images is retained undistorted compared with other current methods, by decreasing the number of antennas. This outcome signifies that the new approach has the advantage of considerably reducing the central processing unit (CPU) time since it only utilizes nearly half the number of antennas.

The main objectives of the presented work are:

- The detection of breast tumors based on (SAR) parameter.
- The development of a modified delay-and-sum (DAS) algorithm to enhance tumor response.
- Combine the modified algorithm and (SAR) parameter to provide high quality image of breast tumor with fast processing.
- Speed up the computing time by employing fewer antennas.

This paper is organized as follows: Section 2 provides an explanation of the simulation set up of the breast-imaging system that is considered in this study. Section 3 describes the confocal algorithm, imaging reconstruction, and process flow of the new approach. Section 4 presents the results and discussions. Finally, the conclusions are presented in Section 5.

## 2. Explanation of The Simulation Setup

**Figure 1:**
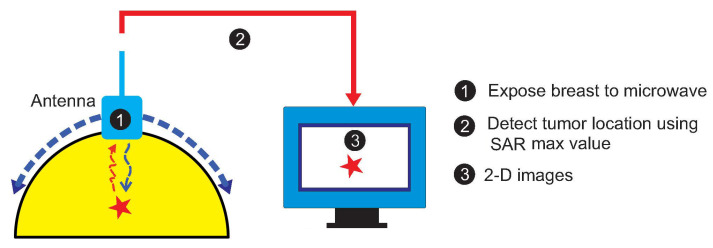
The concept of the considered breast-imaging system.

Figure 1 depicts the concept of the considered breast-imaging system. The schematic diagram is specifically focused on a compact antenna to transmit and receive wideband signals, a human breast phantom modeling, the detection of a tumor insite human breast by determining the SAR maximum values, and data acquisition. The whole system is managed by a personal computer (PC), which is used for signal processing, reconstruction algorithm execution, and image formation.

### 2.1. Breast-Imaging System

Several breast phantom models have been reported in the literature [28,29,30]. In this work, a model of a hemispherical shape with the most common dimensions has been used in all the simulations. The breast model consists of a 50 mm radius hemisphere attached to a 2 mm layer that replicates the skin, filled with air. In most medical imaging literature [31,32,33], the tumor shape is represented as a spherical object. In this work, a spherical 0.5 cm radius tumor is included. This tumor size is chosen based on medical practice as these dimensions represent the limiting case for Stage 1 breast cancer [34], an optimal stage for detection and successful treatment. Dielectric properties used to construct the breast model and tumor are presented in Table 1.

**Figure 2:**
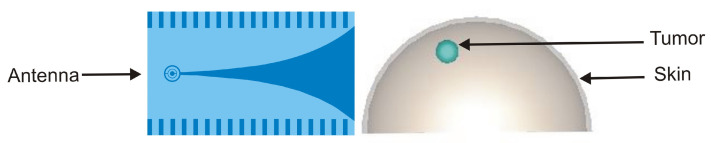
Breast imaging model set up.

Figure 2 illustrates the simulation setup for the considered breast imaging method where the antenna is located at 10 mm distance from the model surface along *Z*-axis for suitable penetration of microwave signal inside the tissue layer, while the tumor coordinates in (mm) are determined at (21, 92, −2). The origin of the coordinate system lies at the center of the hemisphere breast model. The microwave propagation in the breast is described by the finite-difference time domain (FDTD) method.

**Figure 3:**
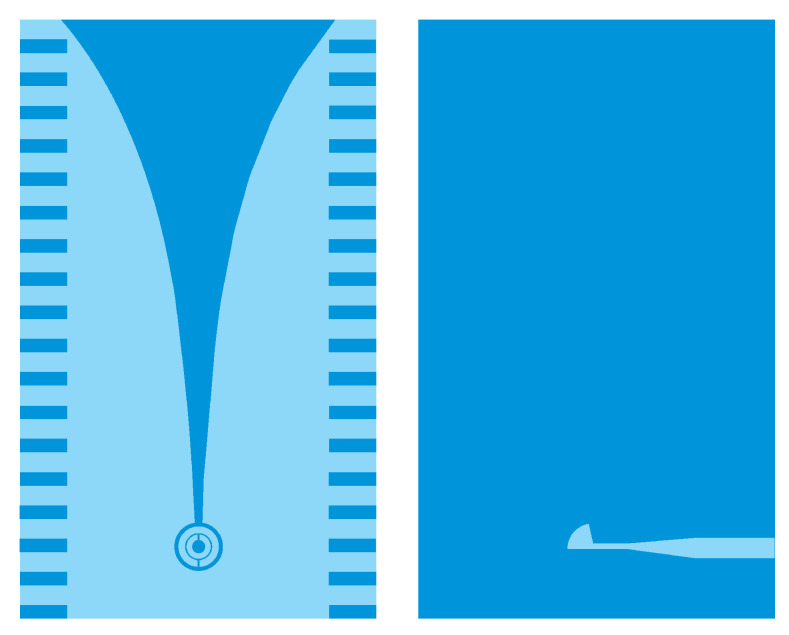
Geometrical structure of the antenna.

In near-field applications such as the UWB microwave imaging system for breast cancer detection, employing an antenna with UWB, high directivity, and moderate gain characteristics is very advantageous. In fact, using a high directional antenna entails the antenna’s power more effectively on the region of interest. The directivity feature effectively increases scattered energy bounced from the tumorous region of the breast tissue. Moreover, the antenna is required to be small, low-cost, and to support a very short pulse transmission with negligible distortion [35]. We therefore used a high directive corrugated Vivaldi antenna with a loaded-SRR structure, which we had developed and designed for radar and medical applications in our laboratories in Tetouan, Morocco and Vigo, Spain. The tapered slot Vivaldi antenna was first proposed by Gibson in 1979, and it has received much attention for possessing striking advantages owing to its ultra-wideband performance, light weight, and high efficiency [36]. It has been adopted in many fields, such as radar systems, microwave imaging system, astronomy, vehicular communication, and remote sensing systems [37]. However, its low directivity is considered as one of drawbacks facing this type of antenna. In this article, we used an enhanced version of a conventional tapered slot Vivaldi antenna (TSVA). The directivity is improved by introducing corrugations periodically on the edges of TSVA. Moreover, the split ring resonator metamaterial is loaded at the circular cavity to increase the gain and providing low return loss characteristics. Figure 3 illustrates the geometrical structure of the antenna. The antenna is designed on the 1.6 mm thick FR4 substrate with permittivity $\epsilon_{r}$ = 4.4. It has an overall size of 53 × 90 mm and radiates at 5.8 GHz corresponding to the ISM band. It exhibits high directional properties and a gain of 6.15 dB at 5.8 GHz.

### 2.2. Proposed Novel Approach for Microwave Imaging System

The target detection in an image is highly sensitive to the positioning of antennas, which guarantees better focusing of the image [38]. In this work, the core concept of the proposed approach is to employ the SAR parameter to determine the optimized positions for the transmitting antennas, which can provide high image quality of the breast tumor. The specific absorption is defined as the measure of the amount of RF energy absorbed by a human body when exposed to the electromagnetic field. It is defined as

(1) $$SAR=\frac{d}{dt}\left(\frac{dW}{dm}\right)=\frac{d}{dt}\left(\frac{dW}{\rho dV}\right)$$

where (dW) is the incremental energy absorbed by an incremental mass (dm) contained in a volume element (dV) of a given mass density ρ. Regarding the electromagnetic energy, SAR can be obtained using the electric field in tissue as

(2) $$SAR=\frac{\sigma {\left|E\right|}^{2}}{\rho }$$

σ is the conductivity (S/m) of the tissue, *E* is the internal electric field (V/m), and ρ is the mass density (Kg/m^3^). According to [27], the difference in the amount of amount being absorbed by the human breast may signify the presence of a tumor. In fact, the cancerous breast model absorbed more energy and registered a higher SAR value compared to the healthier breast model. This research study indicates the utility of the SAR parameter in defining the tumor position. The maximum value of SAR is calculated using CST at 5.8 GHz for tumor detection, adopting a different tumor sizes. CST offers whole-body-averaged and local SAR values. The value is obtained by dividing the total power absorbed in the human body by the full body weight. The results are presented in Table 2.

**Figure 4:**
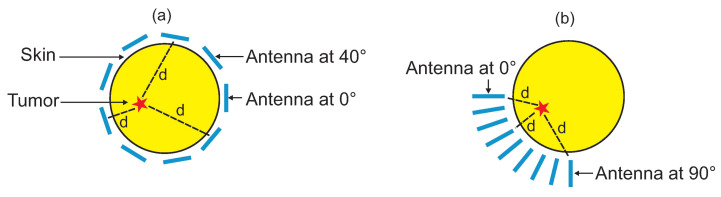
Antenna covering nine positions around the breast using: (**a**) the traditional CMI and (**b**) the proposed approach.

It is demonstrated that the maximum value of SAR actually points to the tumor position regardless the tumor size. In this case, instead of having the Vivaldi antenna scans around the breast for a full 360${}^{\circ }$ (as in the traditional CMI), we only need to replace the antenna at locations close to the tumor with the purpose that the radiated energy is focused in the same area. This antenna relocation leads to a shorter propagation distance *d* and much less degree scan (Figure 4). As a result, stronger signals are obtained to detect the location’s tumor with higher spatial accuracy. Another superiority of this new method is the fast computing time in image reconstruction. When the size of matrices is quite big, the executing process is intensely time consuming. One of the ways to speed up the algorithm of imaging is to decrease the number of samples. In this approach, we manage to accelerate the processing time while maintaining the same number of samples.

A monostatic approach is employed for data acquisition. The collected data are imported into MATLAB and are processed to create a clear image of the breast using the confocal algorithm. There are several programs that can be used for the similar modeling, such as Mathematica and Simulink. However, in our group, we have are in the habit of working with Matlab. Several groups who work on the same topic also use Matlab. That is because of its robustness and efficiency. The antenna is excited with a Gaussian monocycle pulse generated by the CST. It can be expressed as [39]:

(3) $$S\left(t\right)=-\sqrt{e}\cdot \frac{2\pi }{\tau }\cdot \left(t-T_{c}\right)\cdot exp\left(-\frac{1}{2}\cdot {\left(2\cdot \pi \cdot \frac{t-T_{c}}{\tau }\right)}^{2}\right)$$

where $T_{c}$ is the time shift factor, and τ is the impulse width and is equal to $\frac{1}{f_{0}}$ with $f_{0}$ is the center frequency. The Gaussian pulse, given in Equation (3) with the center frequency of 5.8 GHz, allows the signals to propagate a depth of a few centimeters into the breast without excessive attenuation, providing an adequate breast examination. Through repetition, signals are collected and used for image reconstruction.

## 3. Confocal Algorithm and Image Reconstruction

### 3.1. The Detailed Process Flow of the New Approach

**Figure 5:**
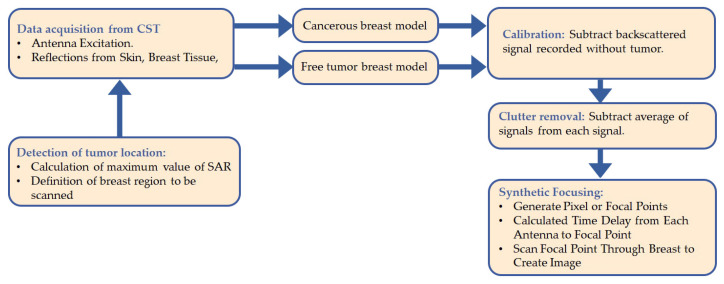
Process flow diagram of the novel approach.

Figure 5 shows the process flow used to create an image of the tumor. At every *x* location, the time domain data of the tumor-free breast model and cancerous breast model were obtained by simulation using the CST Microwave Studio software.

Using a developed MATLAB algorithm, the tumor signature is determined by subtracting the time domain signals received from a tumor-free from those where a tumor exists. However, this tumor response still contains additional unwanted signals such as antenna reverberation. Therefore, to remove clutter, the tumor response is first averaged, and the averaging signal is then subtracted from the tumor response extracted on each antenna location. The signals obtained after clutter removal, called the processed signal, are synthetically focused at a specific point in the breast.

### 3.2. Data Acquisition

After localizing the tumor position using SAR, the Vivaldi antenna is positioned close to the area where the tumor exists, and it is moved to nine different locations arranged in three rows/three columns. The angular separation between each antenna location is 10${}^{\circ }$, leading only to a 90${}^{\circ }$ angle scan on the breast. The data collection procedure is repeated for each antenna position to produce a complete breast image.

XY are the antenna positions defined by row *X* and column *Y* in a grid of 100 mm × 100 mm. The grid is represented as a matrix of 3 × 3. Each (x,y) represents the array response vector of the backscattered signal at a particular position. For nine antenna positions, the matrix is as follows:

(4) $$\left(\begin{array}{ccc} \left(1,1\right) & \left(1,2\right) & \left(1,3\right)\\ \left(2,1\right) & \left(2,2\right) & \left(2,3\right)\\ \left(3,1\right) & \left(3,2\right) & \left(3,3\right) \end{array}\right)$$

Let us represent the tumor-free breast model signals and the cancerous breast model signals by *B* and TB, respectively. Therefore, for *n* = 9 antenna positions, $B_{XY}\left(t\right)$ and $TB_{XY}\left(t\right)$ can be defined as follows:

(5) $$\left(\begin{array}{ccc} B_{11}\left(t\right) & B_{12}\left(t\right) & B_{13}\left(t\right)\\ B_{21}\left(t\right) & B_{22}\left(t\right) & B_{23}\left(t\right)\\ B_{31}\left(t\right) & B_{32}\left(t\right) & B_{33}\left(t\right) \end{array}\right)\left(\begin{array}{ccc} TB_{11}\left(t\right) & TB_{12}\left(t\right) & TB_{13}\left(t\right)\\ TB_{21}\left(t\right) & TB_{22}\left(t\right) & TB_{23}\left(t\right)\\ TB_{31}\left(t\right) & TB_{32}\left(t\right) & TB_{33}\left(t\right) \end{array}\right)$$

### 3.3. Calibration

The first step in image reconstruction is the calibration process. This involves suppressing the response of the $TB_{XY}\left(t\right)$ from the $B_{XY}\left(t\right)$ in order to get an approximate tumor waveform $T_{XY}\left(t\right)$

(6) $$T_{XY}\left(t\right)=TB_{XY}\left(t\right)-B_{XY}\left(t\right)$$

where $T_{XY}\left(t\right)$ represents the tumor signal at time *t* for nine antenna positions and is defined as

(7) $$\left(\begin{array}{ccc} T_{11}\left(t\right) & T_{12}\left(t\right) & T_{13}\left(t\right)\\ T_{21}\left(t\right) & T_{22}\left(t\right) & T_{23}\left(t\right)\\ T_{31}\left(t\right) & T_{32}\left(t\right) & T_{33}\left(t\right) \end{array}\right)$$

### 3.4. Clutter Removal

In order to construct an accurate image, we need to improve the tumor response by suppressing the clutter resulting from the antenna reflection and environment. First, $T_{XY}\left(t\right)$ signals are averaged over the total number of antenna elements *n*. This process is carried out by adding the $T_{XY}\left(t\right)$ signals in a given row of grid (3 × 3) and dividing the result by the total number of antenna in that row *n*. The resultant signals are known as the processed signals $P_{XY}\left(t\right)$ and can be expressed as

(8) $$P_{XY}\left(t\right)=T_{XY}\left(t\right)-A_{X}\left(t\right)$$

where $A_{X}\left(t\right)$ and can be defined as

(9) $$A_{X}\left(t\right)=\frac{\sum_{Y=1}^{n}T_{XY}\left(t\right)}{n}$$

For the overall antenna positions, the processed signals $P_{XY}\left(t\right)$ at location XY and at time *t* can be expanded as

(10) $$\left(\begin{array}{ccc} P_{11}\left(t\right) & P_{12}\left(t\right) & P_{13}\left(t\right)\\ P_{21}\left(t\right) & P_{22}\left(t\right) & P_{23}\left(t\right)\\ P_{31}\left(t\right) & P_{32}\left(t\right) & P_{33}\left(t\right) \end{array}\right)$$

### 3.5. Synthetic Focusing

**Figure 6:**
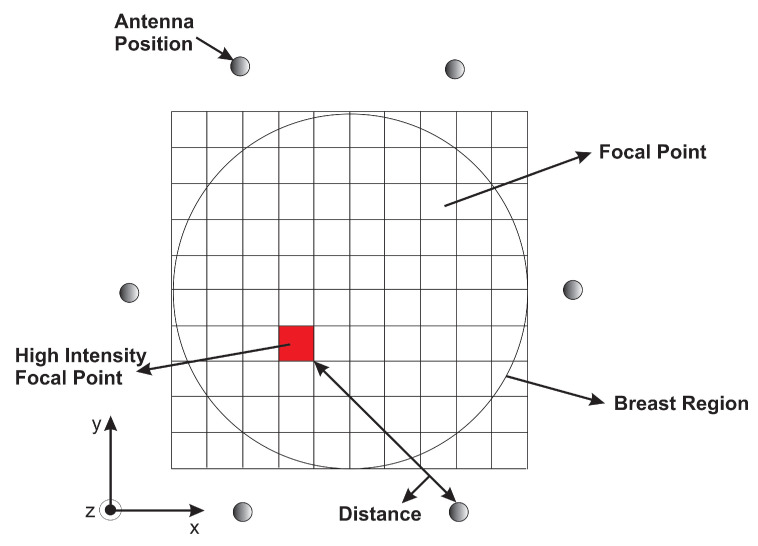
A representation of the monostatic approach.

The processed signals are synthetically focused at a specific point in the breast for image formation. For that, the interested area to be imaged is meshed into a pixel grid as illustrated in Figure 6. Each grid is imagined as a focal point. To generate the pixel points at the $x_{i}$ and $y_{j}$ coordinates, and to obtain a higher resolution for tumor detection, we consider an area of (350 × 350) with sufficient pixels per inch for more focal points instead of considering the area covered by the antenna positions (100 × 100). Therefore, this will allow for high image resolution with a fast computation time. During the focusing, the focal point moves from one position to another within the breast, resulting in spatial beam-forming.

Next, the time required to travel the round trip distance between the focal point $\left(x_{i},y_{j}\right)$ and the antenna position (X,Y) is determined. This travel time, called the round-trip time, relies on the estimated average of the environment permittivity.

The distance(s) of each antenna position (X,Y) to the pixel $\left(x_{i},y_{j}\right)$ was evaluated using the following equation:

(11) $$D_{XY}\left(x_{i},y_{j}\right)=2\sqrt{{\left(X-x_{i}\right)}^{2}+{\left(Y-y_{j}\right)}^{2}+h^{2}}$$

where *h* is the height of the antenna from the skin along the *Z*-axis, representing *Z*-coordinates; $x_{i}$ and $y_{j}$ represent the coordinates of the pixel point; and *X* and *Y* represent the antenna positions. Since the radar approach employed in this study is based on the monostatic radar, the round trip distance is multiplied by 2.

For the antenna position (X,Y), the round trip at pixel $\left(x_{i},y_{j}\right)$ can then be calculated by the following equation:

(12) $$t_{XY}\left(x_{i},y_{j}\right)=\frac{D_{XY}\left(x_{i},y_{j}\right)}{c/\sqrt{\varepsilon_{r}}}$$

where *c* is the speed of light in cm/s, $\varepsilon_{r}$ is the permittivity of the medium (skin in our case), and $t_{XY}\left(x_{i},y_{j}\right)$ is the round trip time from antenna position (X,Y) to pixel $\left(x_{i},y_{j}\right)$.

After calculating the time values for each antenna position (X,Y), the intensity values are generated for each processed signal $P_{XY}\left(t\right)$ at time $t_{XY}\left(x_{i},y_{j}\right)$. These intensities are mapped, and an image of the breast is reconstructed. The intensity of a pixel in the reconstructed image can be expressed as

(13) $$I\left(x_{i},y_{j}\right)={\left[\sum_{X=1}^{r}\sum_{Y=1}^{c}P_{XY}\left(t_{XY}\left(x_{i},y_{j}\right)\right)\right]}^{2}$$

where *r* is the total number of antenna positions in a row, *c* is the total number of antenna positions in a column, and $I(x_{i},y_{j})$ is intensity of pixel values at time $t_{XY}\left(x_{i},y_{j}\right)$.

## 4. Results and Discussion

For image construction, a new MATLAB program is written to image the tumor using the algorithm discussed in previous section besides the program written in [40] for comparison. In the method presented in [40], they used a monostatic antenna configuration. The antenna was moved to 36 positions around the breast, forming a 360 degree scan. I would like to mention here that the signals in both methods were created by the same way. The program involves tumor detection using DAS beamforming algorithm. The imaging results were obtained by processing the recorded waveform at different excitation locations, with different tumor sizes and materials. The power of intensity in Equation (13) was increased for better image resolution and to quantify the presence of any artifact at the location of the tumor inside the breast. This choice has been done after several tests since it has proved to reduce artifacts in [40,41,42].

### 4.1. Size of Tumor

**Figure 7:**
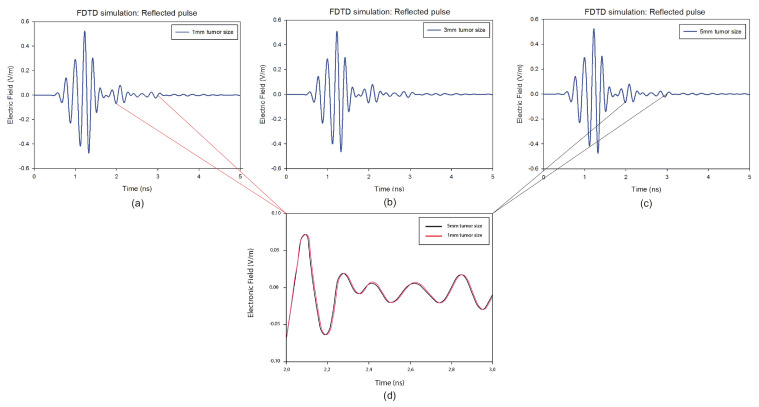
Backscattered responses with different tumor size at a predefined position of the breast model: (**a**) 1 mm, (**b**) 3 mm, (**c**) 5 mm. (**d**) represents a comparison between the responses with the size of 1 mm and the size of 5 mm in the frame of 1 ns.

Figure 7 shows the recorded time-domain voltage waveforms for a particular x position in the presence of 5 mm, 3 mm, and 1 mm radius tumor.

**Figure 8:**
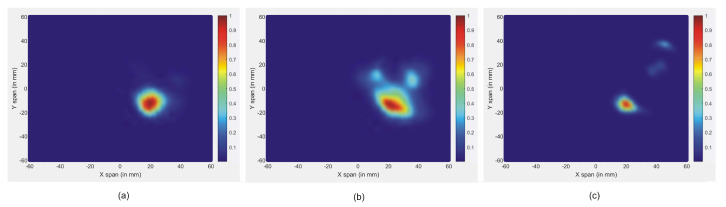
Reconstructed images of different tumor size: (**a**) 5 mm, (**b**) 3 mm, and (**c**) 1 mm.

It is clear that the skin reactions are the same for different tumor sizes, and the tumor information appears within the time intervals 2 ns to 3 ns, which are zoomed in each figure. Figure 8 illustrates the reconstructed images of all different tumor sizes. The proposed approach is able to detect a tumor with a 1 mm radius embedded inside the breast model, while it shows much better imaging results with a 5 mm tumor. For this reason, the 5 mm tumor is chosen as the designed tumor size in the next studies.

### 4.2. Material of Tumor

The efficacy of advancing the microwave breast cancer detection technique depends on the microwave dielectric properties of normal and malignant breast tissues. These dielectric properties determine the interactions of the tissue with the electromagnetic fields. Knowledge of these properties is therefore considered as crucial. In this work, we tested the performance of the new approach by examining several different dielectric properties of tumor models, which were considered in [43]. Table 3 presents the dielectric properties of different tumor models.

**Figure 9:**
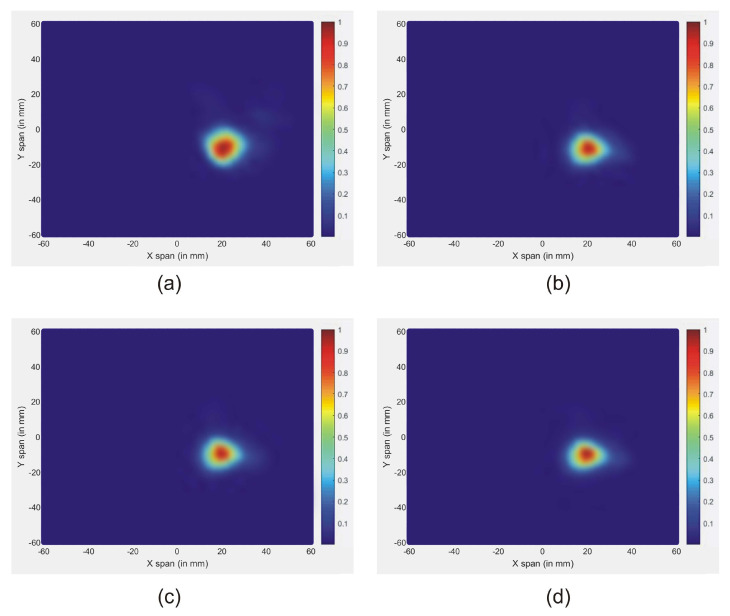
Reconstructed images of different tumor permittivity: (**a**) Tumor 1, (**b**) Tumor 2, (**c**) Tumor 3, and (**d**) Tumor 4.

Figure 9 shows the reconstructed images of different tumor permittivity, located at *x* = 0. It is noted that the effectiveness of the new approach signifies the tumor responses for all the permittivities. In all figures, the maximum scattered energy occurs at the tumor location. This indicates that all tested tumors are clearly detected. Further, the size and location of the tumors are well represented in images.

### 4.3. Comparison with Existing Method

In a multi-static mode, the total number of antennas required for imaging affects the quality of the reconstructed image. In fact, enhanced image quality is achieved when employing a larger number of antennas. However, the use of multiple antennas increases the cost of RF modules, while a low number of antennas fail to obtain the correct targets detection due to smaller scattered data-sets.

**Figure 10:**
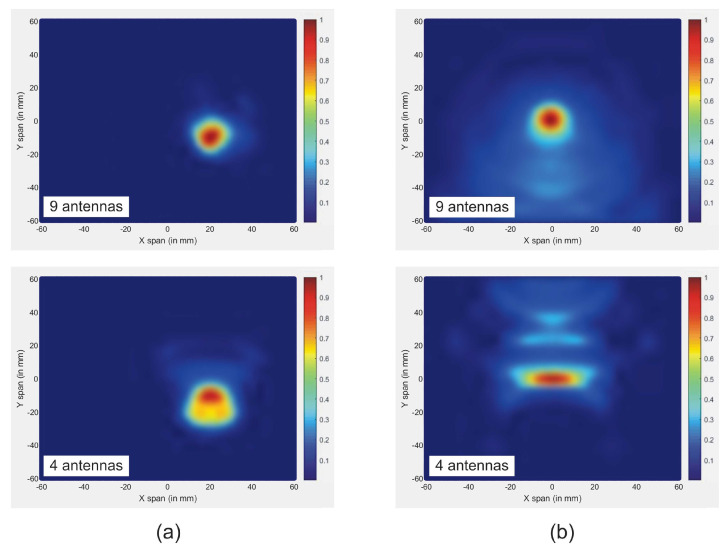
Reconstructed images of tumor for both methods using different number of antennas: (**a**) new approach; (**b**) traditional approach.

**Figure 11:**
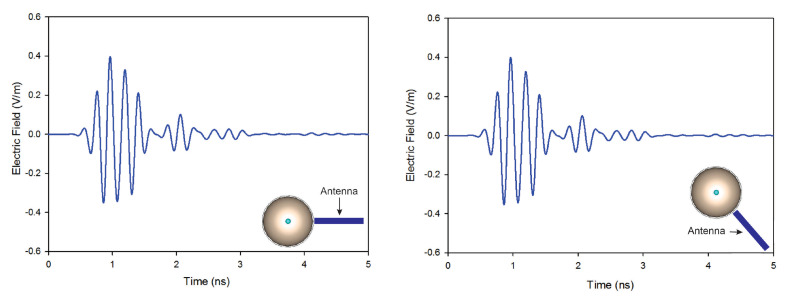
Back-scattered responses at two antenna positions using the conventional method.

**Figure 12:**
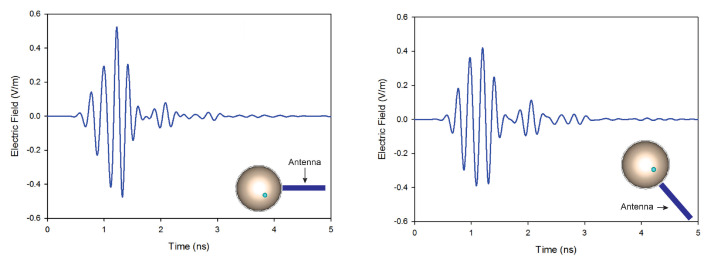
Back-scattered responses at two antenna positions using the proposed method.

In this article, we investigate the influence of number of antennas on the reconstructed images. On the first simulation, nine antennas are used to transmit and receive signals, while on the second only four antennas are used. Figure 10 shows the reconstructed images of both simulations. The results indicate that when the number of antennas used is decreased, the quality of the obtained image is distorted by using a traditional confocal imaging algorithm; however, when the new approach was applied, the position of the tumor was successfully detected using only four antennas (a short processing time). The directive behavior of the antenna with the limited number of antennas used for image construction improves the tumor response and surely decreases interferences. Moreover, the total processing time (for a computer of 3.4 GHz CPU and 6 GB RAM) to generate an image with 4000 samples is 61% less than the method presented in [40], which makes the proposed approach a quasi-real-time. Figure 11 represents the level of intensities at two antenna positions for the conventional method, while Figure 12 illustrates the level of intensities for the proposed approach at the same antenna positions. In the methods presented in [40,42], they opted for a simple case that had the tumor placed in the middle of the breast, which was not always the case. In the proposed method, the tumor was detected using SAR, which allowed us to relocate antennas near the tumor wherever it was and reconstruct its image. In Figure 11 and Figure 12, we wanted to present both methods with different tumor location because if the tumor was placed in the center of the breast like in the other methods, we would fail in the same simple case and obtain the same results. Based on the results of these two simulations, it can be noted that when the antenna is placed near the tumor (the new method), the amplitude of the recorded signal at the antenna is increased. This is due to the strong tumor reflection. On the other hand, when the antenna is placed far from the tumor (existing method), the signal strength is reduced. This is due to the increase in the distance between the antenna and the tumor, resulting in more tissue attenuation during wave propagation. The difference in amplitudes may not be very noticeable since the positions of the tumor in boths methods are very close.

## 5. Conclusions

This paper presented a new method for high-quality image reconstruction based on a specific absorption rate aimed at early breast cancer detection. First, to detect the tumor, the maximum value of SAR was calculated using a high directional Vivaldi antenna. Consequently, we relocated the antenna position near the tumor surface, making the separation distance between them smaller. This action reduced the propagation distance of the RF signals in the image reconstruction operation. The antenna was then moved to nine different positions to transmit short pulses of low power waves into the breast model. The reflected signals were collected to be processed in MATLAB for image reconstruction. Finally, we improved the conventional DAS algorithm by optimizing the distance parameter in the synthetic focusing matrices. The proposed technique was tested and analyzed using several testing scenarios for reconstruction imaging, and different sizes and permittivities of tumors were investigated to prove its effectiveness. The reconstructed images with fewer antennas were also verified. The resulting images were clear enough to indicate the size and the position of the tumor. In addition, when increasing the number of antennas, the image quality remained accurate. These results showed that the proposed method is low-cost; more precise; and faster, 61% faster than the existing method. Although the proposed system yielded excellent results, it has some limitations in detecting multiple tumors. Further work will involve searching for new techniques to be employed in the proposed system in order to detect more than one tumor with a 3-D imaging technique, recovering more accurate dielectric property distribution.

## Figures and Tables

**Table 1:** Dielectric property and conductivity of breast and tumor model.

	Conductivity σ (S/m)	The Relative Permittivity $\varepsilon_{r}$
Skin	1.1	37
tumor	1.2	50

**Table 2:** Maximum value of SAR for different tumor sizes.

Tumor Size (mm)	Max Value of SAR (W/KG)	Max at (*x*, *y*, *z*)
5	44,367	(19.05, −13.64, 14.44)
3	22,445	(21.31, −13.39, 15.76)
1	2139	(20.19, −11.53, 13.98)

**Table 3:** Dielectric properties of different tumor models.

	Permittivity $\varepsilon_{r}$	Conductivity σ (S/m)
Tumor 1	50	1.2
Tumor 2	62.77	1.66
Tumor 3	54	0.7
Tumor 4	55.1	0.79

## Data Availability

Not applicable.

## Abbreviations

The following abbreviations are used in this manuscript:

CMI	Confocal microwave imaging
SAR	Specific absorption rate
DAS	Delay and sum
CMI	Confocal microwave imaging
DI	Data-independent
DA	Data-adaptive
UWB	Ultra-wide band
FDTD	Finite-difference time domain
PC	Personal computer
CPU	Central processing unite
